# Association between *Mycoplasma genitalium* infection and HIV acquisition among female sex workers in Uganda: evidence from a nested case–control study

**DOI:** 10.1136/sextrans-2013-051467

**Published:** 2014-03-31

**Authors:** Judith Vandepitte, Helen A Weiss, Justine Bukenya, Nassim Kyakuwa, Etienne Muller, Anne Buvé, Patrick Van der Stuyft, Richard J Hayes, Heiner Grosskurth

**Affiliations:** 1 MRC/UVRI Uganda Research Unit on AIDS, Entebbe, Uganda; 2 MRC Tropical Epidemiology Group, London School of Hygiene and Tropical Medicine, London, UK; 3 Centre for HIV and Sexually Transmitted Infections, National Institute for Communicable Diseases, National Health Laboratory Service, Johannesburg, South Africa; 4 Prince Leopold Institute of Tropical Medicine, Antwerp, Belgium; 5 University of Ghent, Ghent, Belgium

**Keywords:** AFRICA, EPIDEMIOLOGY (CLINICAL), GENITAL TRACT INFECT, HIV WOMEN, M GENITALIUM

## Abstract

**Objectives:**

Cross-sectional studies have shown a strong association between *Mycoplasma genitalium* and HIV infections. We previously reported that in a cohort of female sex workers in Uganda, *M genitalium* infection at baseline was associated with HIV seroconversion. Here we examine the temporal association between the *M genitalium* infection status shortly before HIV seroconversion and HIV acquisition.

**Methods:**

A nested case-control study was conducted within a cohort of women at high risk for HIV in Kampala. Cases were those of women acquiring HIV within 2 years of enrolment. For each of the 42 cases, 3 controls were selected from women HIV negative at the visit when the corresponding case first tested HIV seropositive. The association between HIV acquisition and *M genitalium* infection immediately prior to HIV testing was analysed using conditional logistic regression.

**Results:**

There was weak evidence of an association between *M genitalium* infection and HIV acquisition overall (crude OR=1.57; 95% CI 0.67 to 3.72, aOR=2.28: 95% CI 0.81 to 6.47). However, time of *M genitalium* testing affected the association (p value for effect-modification=0.004). For 29 case-control sets with endocervical samples tested 3 months prior to the first HIV-positive result, *M genitalium* infection increased the risk of HIV acquisition (crude OR=3.09; 95% CI 1.06 to 9.05, aOR=7.19; 95% CI 1.68 to 30.77), whereas there was little evidence of an association among the 13 case-control sets with samples tested at an earlier visit (crude OR=0.30: 95% CI 0.04 to 2.51; aOR=0.34; 95% CI 0.02 to 5.94).

**Conclusions:**

Our study showed evidence of a temporal relationship between *M genitalium* infection and HIV acquisition that suggests that *M genitalium* infection may be a co-factor in the acquisition of HIV infection.

*Mycoplasma genitalium* infection is an emerging sexually transmitted infection (STI). One-third of nongonococcal nonchlamydial urethritis in men is caused by *M genitalium* infection,^1^ and in women, there is growing evidence that the infection is associated with cervicitis, pelvic inflammatory disease and reproductive health sequelae, although further research is needed to provide conclusive evidence.^2^

*M genitalium* infection is common, especially in HIV-positive individuals where the prevalence ranges between 5% and 33%.^3–8^ A systematic review and meta-analysis found a twofold association between prevalent *M genitalium* and HIV infection (OR=2.01; 95% CI 1.44 to 2.79), with an even stronger association among the nine included studies from sub-Saharan Africa (OR=2.57; 95% CI 2.05 to 3.22).^3^ We previously reported that among female sex workers (FSW) in Kampala, the prevalence of *M genitalium* infection was 18% in HIV-positive and 12% in HIV-negative women (adjusted OR 1.64, 95% CI 1.12 to 2.41, p=0.01).^9^

The temporal relationship between *M genitalium* infection and HIV infection can only be established through longitudinal studies, and so far, there have been very few such studies. Among FSW in Kenya, HIV-positive women had a more than twofold increased risk of acquiring *M genitalium* infection compared with HIV-negative women (adjusted HR 2.17; 95% CI 1.27 to 3.71, p=0.01).^10^ A prospective study among women attending family planning services in Zimbabwe and Uganda found that the risk of acquiring HIV infection was more than twice higher in women infected with *M genitalium* prior to HIV detection (adjusted OR=2.42; 95% CI 1.01 to 5.80).^11^ In our cohort of women with high-risk behaviour in Kampala, women diagnosed with *M genitalium* infection at the baseline visit had a more than twofold higher risk of HIV seroconversion during follow-up time than those who were free of *M genitalium* infection (adjusted HR=2.28, 95% CI 1.15 to 4.52).^12^ The latter finding prompted us to conduct a nested case-control study to examine the *M genitalium* infection status shortly before seroconversion in cases and at an equivalent time-point in matched controls in order to investigate the temporal association between *M genitalium* infection and subsequent HIV acquisition.

## Methods

### Study population and study procedures

Between April 2008 and April 2009, a cohort of 1027 women involved in high-risk sexual behaviour was recruited in Kampala. The establishment of this cohort and the study procedures have been reported previously.^12^ ^13^ In brief, self-reporting FSW and women employed in entertainment facilities located in red-light areas were enrolled and invited to attend 3-monthly visits at the study clinic in Kampala. At every visit, women were interviewed about socio-demographic characteristics, risk behaviour, reproductive health history and symptoms of STIs. Blood was tested for HIV, herpes simplex virus type 2 (HSV2) and syphilis. Two endocervical specimens were collected, one for the immediate diagnosis of gonococcal and chlamydial infection and one for later diagnosis of *M genitalium* infection. One high vaginal specimen was inoculated for culture of *Trichomonas vaginalis*, another for the detection of bacterial vaginosis and *Candida* infection. Symptomatic STIs were treated syndromically and asymptomatic STIs were treated as soon as laboratory results became available. From the second year of follow-up onwards, due to financial constraints, genital sample collection was reduced from 3-monthly to 6-monthly, whereas blood tests and epidemiological data were collected every 3 months, as before. As testing for *M genitalium* infection was done some 2–3 years after sample taking, none of the women were specifically treated for *M genitalium* infection at the time of the visit.

The current analyses use data up to 31st March, 2011. Cases were women who were HIV negative at enrolment and acquired HIV before the end of follow-up. For each case, three controls, individually matched by age group (15–24, 25–34, >35 years), were selected randomly from women who were HIV negative after a similar length of time in the cohort as the cases. For instance, if a case was found to be HIV infected at the 3rd quarterly visit, controls were selected among the women who were still HIV negative at their 3rd quarterly visit. *M genitalium* testing was performed on endocervical samples collected at the last HIV-negative visit for cases and at equivalent visits for controls. If no sample was collected at the relevant visit for the case or for any of their controls, the most recent available sample from an earlier visit was taken for the case and all three controls. For instance, if HIV seroconversion was detected at the month 9 visit and no sample was available at the month 6 visit either from the case or from any of their selected controls, the sample at the month 3 visit was tested for *M genitalium* infection for the case and all three controls.

### Laboratory procedures

Serum specimens were first tested for HIV-1 infection using a rapid test, the Abbott Determine HIV-1/2. Positive samples were confirmed by two ELISA tests (Vironostika Uniform II plus O and Murex HIV 1.2.O). Antibodies against HSV-2 were tested for using an HSV-specific IgG ELISA (Kalon Biologicals Ltd). Syphilis was tested with the Rapid plasma reagin (RPR) assay and the Treponema pallidum Hemagglutination assay (TPHA) (Biotec Laboratories Ltd). *Neisseria gonorrhoeae* and *Chlamydia trachomatis* were diagnosed using the Amplicor PCR test (Roche Diagnostic Systems Inc., Branchburg, New Jersey) and *Trichomonas vaginalis* was detected using the commercial culture kit (InPouch TV, BioMed Diagostics, White City, Oregon, USA). Microscopy on a Gram-stained vaginal specimen was performed for bacterial vaginosis (using Nugent's criteria) and *Candida* infection. All these tests were performed at the laboratories of the MRC/UVRI Research Unit in Entebbe.

The endocervical specimens collected for the *M genitalium* studies were stored at −20°C at the MRC/UVRI laboratories within 12 h of collection. Testing for *M genitalium* infection was carried out at the Centre for HIV and STIs, National Health Laboratory Service in Johannesburg, using a commercially available Real-TM PCR assay (Sacace Biotechnologies, Como, Italy). Details of this PCR test were reported previously.^9^

### Statistical analysis

Data were double entered in Access and analysed using STATA V.11.0 (Stata Inc., College Station, Texas, USA). The OR and 95% CI for the association between HIV acquisition and *M genitalium* infection were estimated using conditional logistic regression to allow for the individual matching of cases and controls. A multivariable analysis was conducted to adjust the association of HIV acquisition and *M genitalium* infection for confounding, with factors retained in the final model if their inclusion changed the OR for the association of HIV and *M genitalium* infections substantially (>20%) due to the relatively small number of events.^14^ For time-dependent factors, the data were from the visit for which the *M genitalium* result was available. For non-time-dependent factors, baseline data were used.

Although participants were scheduled at 3-monthly visits, endocervical samples were not always available from the visit 3 months prior to HIV detection either because the participant did not attend or no genital samples had been collected. Therefore, a multivariable analysis stratified by time of *M genitalium* testing (3 months vs ≥6 months prior to HIV seroconversion) was conducted to investigate the relationship between HIV seroconversion and time of *M genitalium* infection.

### Ethical considerations

Written informed consent, or witnessed fingerprint consent in case of illiteracy, was obtained from all participants before enrolment. The study was approved by the Science and Ethics Committee of the Ugandan Virus Research Institute, by the Uganda National Committee for Science and Technology and by the ethics committee of the London School of Hygiene and Tropical Medicine.

## Results

Among the 1027 participants, 646 were HIV-negative at enrolment (HIV-prevalence 37%). Between 1 April 2008 and 31 March 2011, 42 women acquired HIV, giving an HIV incidence rate of 3.66/100 person years (pyr) (95% CI 2.71 to 4.96/100 pyr).

The study population for the nested case-control study consisted of these 42 cases and 126 matched controls. For 29 case-control sets, *M genitalium* testing was carried out at the visit 3 months prior to the first HIV-positive test result, while for the remaining 13 sets, testing was done at an earlier visit (nine sets at 6 months, two sets at 9 months, one set at 15 months and one set at 18 months prior to HIV detection).

The 42 cases and 126 controls were similar with respect to marital status, level of education, income related to sex work, and sexual risk behaviour in terms of number of sexual partners and condom use with paying clients (table 1). Cases were more likely to report use of illicit drugs and alcohol during follow-up and to be classified as problem drinkers at enrolment than their corresponding controls. HSV2, *N gonorrhoeae* and bacterial vaginosis were more prevalent at the visit prior to the HIV positive test among cases than among controls and this difference was statistically significant. *M genitalium* infection was detected in 10 (24%) of cases and in 21 (17%) of controls. On univariable analysis, there was some evidence of an association between prior *M genitalium* infection and HIV acquisition (OR=1.57; 95% CI 0.66 to 3.72). The association became stronger after adjusting for confounders (adjusted OR=2.28; 95% CI 0.81 to 6.47) (table 2).

**Table 1:** Characteristics of HIV seroconverters (cases) and their age-matched controls

	Total study population		
	Cases n (%)	Controls n (%)	Crude OR (95% CI)
	N=42	N=126	
*Socio demographic factors*			
Age (matching variable)			
14–24 years	26 (62%)	78 (62%)	–
25–34 years	15 (36%)	45 (36%)	–
>35 years	1 (2%)	3 (2%)	–
Marital status*			
Formerly married	29 (69)	71 (56)	1
Currently married	5 (12)	23 (18)	0.52 (0.17 to 1.54)
Single	8 (19)	32 (25)	0.59 (0.23 to 1.52)
Level of education†			
Primary completed or higher	24 (57)	73 (58)	1
Primary uncompleted or no education	18 (43)	53 (42)	1.03 (0.53 to 2.01)
Source of income*			
Sex work only	15 (36)	26 (21)	1
Sex work and other	22 (52)	87 (69)	0.41 (0.18 to 0.94)
No sex work	5 (12)	13 (10)	0.61 (0.17 to 2.19)
*Behavioral factors*			
Alcohol use defined by CAGE score†			
Not drinking/not problem drinking	11 (26)	62 (49)	1
Problem drinking	31 (74)	64 (51)	2.90 (1.31 to 6.42)

	Total study population		
	Cases n (%)	Controls n (%)	Crude OR (95% CI)
No. of sexual partners in last month*			
<5	17 (40)	66 (52)	1
5–19	11 (26)	29 (23)	1.42 (0.59 to 3.44)
20–49	7 (17)	22 (18)	1.33 (0.46 to 3.82)
50+ or can't remember	7 (17)	9 (7)	2.96 (0.96 to 9.15)
Paying clients in last month*			
No	8 (19)	22 (17)	1
Yes	34 (81)	104 (83)	0.90 (0.36 to 2.23)
Condom use with paying clients in last month*,‡			
Consistent	14 (41)	51 (49)	1
Inconsistent	20 (59)	53 (51)	1.39 (0.62 to 3.11)
Intravaginal cleansing in past 3 months*			
Cleansing using soap	19 (45)	62 (49)	1
Cleansing using water only	18 (43)	60 (48)	0.96 (0.47 to 1.97)
No cleansing	5 (12)	4 (3)	3.69 (0.95 to 14.25)
*Reproductive health*			
Currently pregnant*			
No	38 (90)	111 (88)	1
Yes	4 (10)	15 (12)	0.78 (0.25 to 2.48)
Use of hormonal contraceptives in past 3 months*,§			
None	19 (50)	64 (58)	1
Oral	5 (13)	13 (12)	1.29 (0.41 to 4.03)
Injectable	14 (37)	34 (31)	1.40 (0.61 to 3.21)
*Reproductive tract infections*			
Mycoplasma genitalium*			
Negative	32 (76)	105 (83)	1
Positive	10 (24)	21 (17)	1.57 (0.67 to 3.72)
Herpes simplex virus type 2 serology*			
Negative	3 (7)	48 (38)	1
Positive	39 (93)	78 (62)	7.89 (2.32 to 26.8)
Syphilis*			
RPR−TPHA−	31 (74)	102 (81)	1
RPR−TPHA+	2 (5)	11 (9)	0.58 (0.12 to 2.70)
RPR+TPHA+	9 (21)	13 (10)	2.15 (0.88 to 5.27)
Neisseria gonorrhoeae*,¶			
Negative	25 (76)	95 (96)	1
Positive	8 (24)	4 (4)	6.00 (1.81 to 19.92)
Chlamydia trachomatis*,¶			
Negative	30 (91)	97 (98)	1
Positive	3 (9)	2 (2)	7.24 (0.73 to 72.04)
Trichomonas vaginalis*,**			
Negative	29 (83)	87 (86)	1
Positive	6 (17)	14 (14)	1.00 (0.31 to 3.26)
Candida albicans*,¶			
Negative	32 (97)	92 (93)	1
Positive	1 (3)	7 (7)	0.40 (0.05 to 3.46)
Bacterial vaginosis*,¶			
Negative/intermediate	8 (24)	55 (56)	1
Positive	25 (76)	44 (44)	3.97 (1.55 to 10.19)

*At same visit as *M genitalium* was tested.

†At baseline visit.

‡Among women with paying clients in past month.

§Among not pregnant women.

¶Missing for 9 cases and 27 controls.

**Missing for 7 cases and 25 controls.

RPR, Rapid plasma regain; TPHA, Treponema pallidum Hemagglutination assay

**Table 2:** Association between prior infection with *Mycoplasma genitalium* and HIV seroconversion

	Cases n (%)	Controls n (%)	Crude OR (95% CI)	Adjusted OR* (95% CI)
Total study population				
*M genitalium*			p=0.30	p=0.10
Negative	32 (76)	105 (83)	1	1
Positive	10 (24)	21 (17)	1.57 (0.67 to 3.72)	2.28 (0.81 to 6.47)
*M genitalium* tested at visit 3 months prior to first HIV-positive result				
*M genitalium*			p=0.04	p=0.002
Negative	20 (69)	75 (86)	1	1
Positive	9 (31)	12 (14)	3.09 (1.06 to 9.05)	7.19 (1.68 to 30.77)
*M genitalium* tested at visit >3 months prior to first HIV-positive result				
*M genitalium*			p=0.27	p=0.38
Negative	12 (92)	30 (77)	1	1
Positive	1 (8)	9 (23)	0.30 (0.04 to 2.51)	0.34 (0.02 to 5.94)

*Adjusted for problem drinking at baseline and source of income; p value for effect-modification with time=0.01.

The time of *M genitalium* testing affected the observed association with HIV acquisition (p value for effect-modification=0.004). Therefore, the association was re-evaluated separately in case-control sets with and without *M genitalium* test results for the visit prior to HIV seroconversion. For the 29 case-control sets with endocervical samples tested 3 months prior to HIV detection (or equivalent visit for controls), there was evidence that *M genitalium* increased the risk of HIV acquisition (crude OR=3.09; 95% CI 1.06 to 9.05, adjusted OR=7.19; 95%CI 1.68 to 30.77), whereas for the 13 case-control sets with endocervical samples tested at an earlier visit, there was no evidence of association with HIV acquisition (crude OR=0.30; 95%CI=0.04 to 2.51; adjusted OR=0.34; 95%CI 0.02 to 5.94) (table 2).

## Discussion

Our study investigated the temporal relationship between pre-existing *M genitalium* infection and HIV acquisition among women at high risk for HIV in Kampala. We found evidence that women diagnosed with *M genitalium* infection were at threefold higher odds of acquiring HIV during the 3 months following *M genitalium* detection, whereas there was little evidence of an association among the 13 case-control sets with samples tested at an earlier visit.

Our results are consistent with the results of the case-control study of Mavedzenge *et al.,* which had similar time intervals between *M genitalium* and HIV tests (adjusted OR=2.42; 95% CI 1.01 to 5.80).^11^ The difference in strength of association between the studies may be due to chance, may reflect the difference in study populations (high risk vs general population) or may be due to residual confounding in either study.

The association with HIV acquisition is biologically plausible. After inoculation of *M genitalium* in the urogenital tract, bacterial toxins disrupt the mucosal barrier.^15^ More importantly, the host cell responds to the infection with a prominent inflammatory reaction, which triggers cytokine production and increases presence and activation of HIV susceptible cells.^16–18^

The association was affected by the time between *M genitalium* testing and HIV acquisition. The most plausible explanation is that earlier *M genitalium* infection may have cleared spontaneously by the time the woman was exposed to HIV. We reported earlier that in our cohort, among the women diagnosed with *M genitalium* infection at enrolment, 55% spontaneously cleared the infection within 3 months and 83% within 6 months.^19^

Although *M genitalium* infection was not specifically treated at the time of visit, the infection may have cleared because of treatment with antibiotics administered for other STIs or health conditions, and this may have been differential in the two time groups. However, only 14% of the women with recent *M genitalium* infection and none of those with earlier *M genitalium* infection in our study had records showing that they had received treatment likely to be effective against *M genitalium* (doxycycline, ciprofloxacin or erythromycin).

An alternative hypothesis for the discrepancy between both time groups is that the disruptive and inflammatory processes caused by mycoplasma infections may be more abundant during the acute stage of the infection and become less important later on. Due to financial constraints, we were not able to test for *M genitalium* infection at all consecutive visits prior to HIV seroconversion, and so it was not possible to disentangle whether the detected *M genitalium* infections were either early (acute) or rather longer lasting persistent infections.

Limitations of our study were (i.) the small sample size, resulting in wide CIs for the estimated association and (ii.) the large number of potential confounding factors compared with the small number of cases (42). Regression models should be used with a minimum of 10 outcome events per predictor variable.^14^ Therefore, it was decided to include only the factors with the strongest confounding effect (source of income, alcohol use defined by CAGE, HSV-2 infection), besides the matching variable age, to keep the robustness of the regression model intact.

However, the magnitude of the point estimate after adjustment for confounding is compelling, and calls for larger longitudinal studies to confirm the findings.

In conclusion, our findings suggest that *M genitalium* infection may be a co-factor in HIV acquisition. The consistent results obtained from low-11 and high-risk women in a similar setting lend support to the generalisability of the findings. Control measures for *M genitalium* infection should be considered, especially in populations at high risk for HIV.

**Key messages**

- Infection with *Mycoplasma genitalium* immediately prior to HIV seroconversion increased the risk of HIV acquisition among Ugandan female sex workers.
- There was little evidence of an association between *M genitalium* infection and HIV acquisition if *M genitalium* was tested ≥6 months prior to time of first HIV test.
- Screening for and treatment of *M genitalium* infection should be considered as part of the HIV prevention strategy, especially in populations at high risk.
